# Modulation of oxidation-related immune markers by lipid-lowering medications in individuals with elevated lipoprotein(a)

**DOI:** 10.1007/s00392-026-02896-5

**Published:** 2026-03-23

**Authors:** Amalia Despoina Koutsogianni, Fotios Barkas, Constantinos Tellis, Alexandros Tselepis, George Liamis, Sotirios Tsimikas, Evangelos Liberopoulos

**Affiliations:** 1 Department of Internal Medicine, Faculty of Medicine, School of Health Sciences, University of Ioannina, Ioannina, Greece; 2 Department of Chemistry, Division of Organic Chemistry and Biochemistry, School of Natural Sciences, University of Ioannina, Ioannina, Greece; 3 Atherothrombosis Research Center, Laboratory of Biochemistry, Department of Chemistry, School of Natural Sciences, University of Ioannina, Ioannina, Greece; 4 Division of Cardiovascular Medicine, Sulpizio Cardiovascular Center, University of California San Diego, 9500 Gilman Dr, La Jolla, San Diego, CA USA; 5 1st Propedeutic Department of Medicine and Diabetes Center, School of Medicine, National and Kapodistrian University of Athens, Laiko General Hospital, Athens, Greece

**Keywords:** Lipoprotein(a), Autoantibodies, Oxidative stress, Statins, Ezetimibe, PCSK9 inhibitors

## Abstract

**Background:**

Oxidative modification of apolipoprotein B-100 (apoB) containing particles and subsequent immune responses contribute to the pathogenesis of atherosclerosis. Circulating IgG and IgM apoB-containing immune complexes (apoB-IC) and autoantibodies to a malondialdehyde mimotope (anti-MDA-mimotope) serve as biomarkers of oxidative stress and immune activation in atherosclerotic cardiovascular disease. Elevated lipoprotein(a) [Lp(a)] is associated with increased oxidative burden and immune activation.

**Purpose:**

To investigate the effect of lipid-lowering medications on IgG and IgM apoB-IC and IgG and IgM autoantibodies to an MDA-mimotope in individuals with elevated lipoprotein(a) [Lp(a)] concentrations.

**Methods:**

In this prospective study, patients (*n* = 70) with Lp(a) levels ≥ 75 nmol/L were assigned to 3 treatment regimens according to current guidelines: high-intensity statin monotherapy (*n* = 28), ezetimibe added to high-intensity statin (*n* = 31) and proprotein convertase subtilisin/kexin type 9 inhibitor (PCSK9i) added to high-intensity statin plus ezetimibe (*n* = 11). IgG and IgM apoB-IC and IgG and IgM anti-MDA-mimotope were measured at baseline and 3 months after treatment initiation.

**Results:**

Patients had a mean age of 51 ± 15 years and 40% were male. Significant reductions in IgG apoB-IC levels were observed following treatment with high-intensity statins, add-on ezetimibe and add-on PCSK9i (by 18.3%, 17.5% and 25.5%, respectively, all *p* < 0.05). No significant changes in IgM apoB-IC, or IgG and IgM anti-MDA-mimotope levels were observed in any treatment group.

**Conclusions:**

In individuals with Lp(a) levels ≥ 75 nmol/L, high-intensity statins, add-on ezetimibe and add-on PCSK9i reduced IgG apoB-IC but did not affect IgM apoB-IC, or IgG and IgM anti-MDA-mimotope levels. The clinical significance of these findings warrants further investigation.

**Graphical Abstract:**

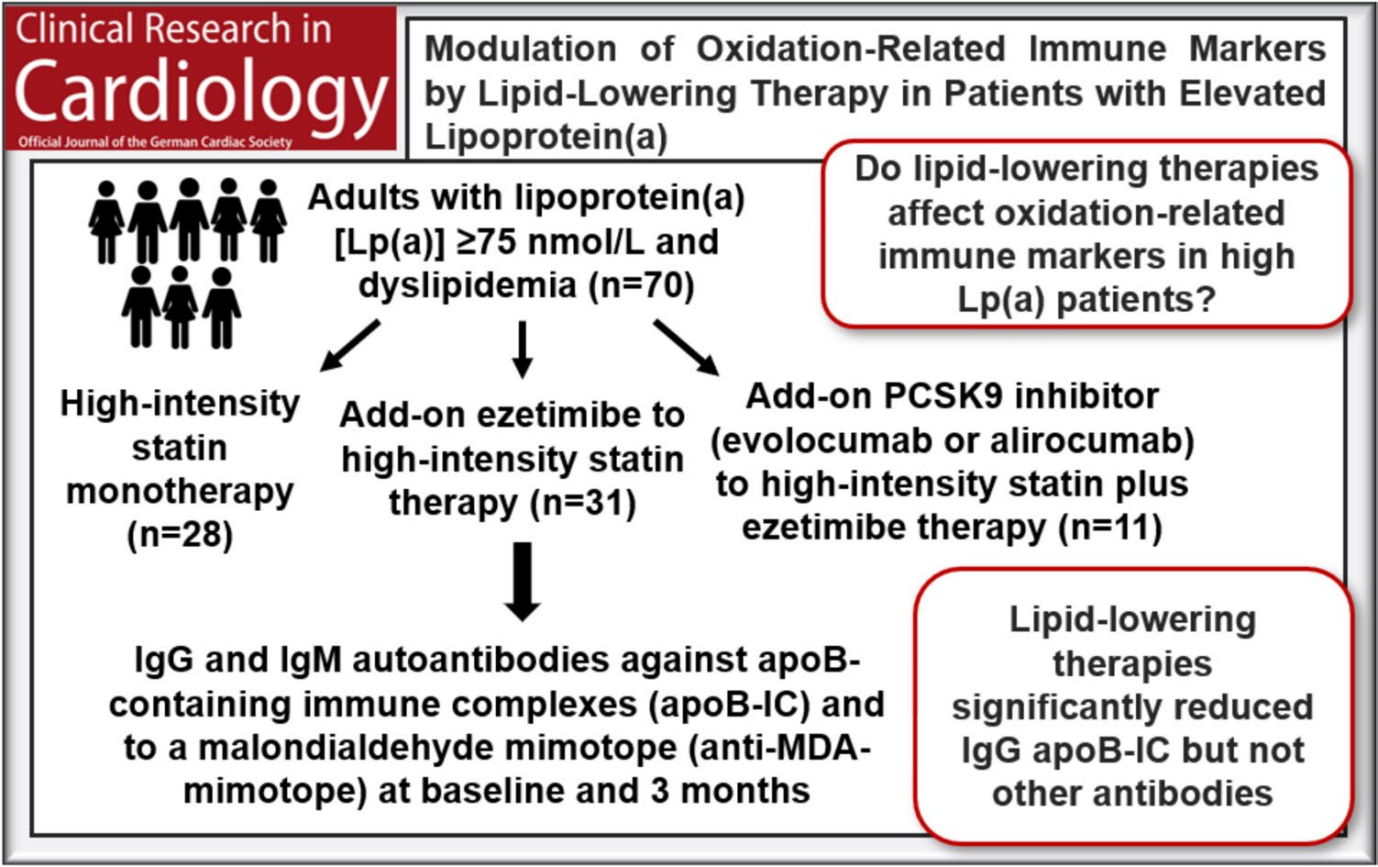

## Introduction

Atherosclerosis is characterized by chronic inflammation and oxidative modification of lipoproteins, particularly low-density lipoprotein (LDL) [1]. Among oxidative modifications, malondialdehyde (MDA) is a highly reactive byproduct of lipid peroxidation formed during the oxidative degradation of polyunsaturated fatty acids that react with peptides, including apolipoprotein B-100 (apoB), forming MDA-apoB adducts or neoepitopes [2, 3]. These MDA-apoB adducts and neoepitopes are recognized as non-self by the immune system, thereby triggering humoral immune responses, including the production of IgG and IgM autoantibodies against MDA-modified epitopes [2]. MDA-modified apoB (MDA-apoB) is considered an important biomarker of oxidative stress, inflammation, endothelial dysfunction, immunogenicity and atherogenicity in atherosclerotic cardiovascular disease [4–11]. Autoantibodies produced by these modifications can be measured as apoB-containing immune complexes (IgG and IgM apoB-IC) as well as autoantibodies targeting an MDA-modified LDL, which can be detected using an MDA-peptide mimotope mimicking oxidation-specific epitopes on apoB (IgG and IgM anti-MDA-mimotope) [12]. Of interest, elevated IgG autoantibodies against MDA-apoB, especially against MDA-modified peptide epitopes such as p45 and p210, have been linked to incident cardiovascular events and plaque instability [9, 13–15].

Elevated lipoprotein(a) [Lp(a)] concentrations are associated with increased oxidative burden and immune activation, which may predispose to enhanced apoB modification and autoantibody generation [16–18]. Indeed, Lp(a) carries a disproportionately high content of oxidized phospholipids (OxPLs), particularly on its apolipoprotein(a) moiety, which colocalizes with apoB on the LDL-like particle [16–18]. OxPLs on Lp(a) serve as potent epitopes for immune recognition, leading to the generation of circulating IgG and IgM autoantibodies targeting oxidation-specific epitopes, including MDA-apoB and mimotopes of oxidized LDL (oxLDL)[16–18]. These immunogenic features may contribute to the proatherogenic and proinflammatory properties of Lp(a) [16–18].

Although high-intensity statins, ezetimibe and proprotein convertase subtilisin/kexin type 9 inhibitors (PCSK9i) are effective in lowering LDL-C and apoB concentrations [19], their effect on oxidized forms of apoB and immune responses remain less well understood. To our knowledge, no study has evaluated how these treatments impact IgG and IgM autoantibodies against apoB-IC or MDA-mimotope epitopes either in the general population or in individuals with elevated Lp(a) concentrations.

Thus, we undertook the present study to investigate the effect of high-intensity statin treatment, add-on ezetimibe, and add-on PCSK9i therapy on IgG and IgM autoantibodies to apoB-IC as well as IgG and IgM autoantibodies against an MDA-mimotope in patients with Lp(a) ≥ 75 nmol/L.

## Material and methods

### Study population

Study design details have been previously published [20]. Briefly, this was a prospective study including consecutive adult patients with Lp(a) levels ≥ 75 nmol/L attending the Outpatient Lipid Clinic of the University Hospital of Ioannina. Patients were non-randomly allocated to 3 treatment groups according to the national guidelines for the management of dyslipidemias [21]. All study participants were of Caucasian origin. We included a group of naïve patients who received high-intensity statin monotherapy, a group of patients on stable high-intensity statin treatment who received add-on ezetimibe and a group of patients on stable high-intensity statin treatment plus ezetimibe who received add-on PCSK9i (evolocumab or alirocumab). The patients were on high-intensity statin treatment and high-intensity statin treatment plus ezetimibe for at least 3 months before adding ezetimibe or a PCSK9i, respectively. High-intensity statin treatment included rosuvastatin 20–40 mg or atorvastatin 40–80 mg [21]. Follow-up duration was 3 months.

### Clinical and laboratory assessments

Venous blood samples were obtained both into sterile Vacutainer-SST II advance tubes (Becton–Dickinson, Plymouth, UK) and into a vacutainer containing potassium EDTA, in the morning after 12 h fasting. Serum and plasma samples were stored at −80 °C.

Lp(a) was measured using a validated, isoform independent enzyme-linked immunosorbent assay (ELISA) traced to WHO/IFCC reference material SRM 2B as previously described, which ensures accurate measurement of Lp(a) levels irrespective of the diverse isoform sizes, overcoming limitations of earlier techniques [22].

### Determination of IgG, IgM autoantibodies titers

Plasma IgG and IgM autoantibodies apoB-IC and MDA-mimotope peptides were quantified using validated chemiluminescent ELISAs adapted from previously published protocols, based on monoclonal capture antibodies and detection of immunoglobulin isotype binding [7, 23–27]. Internal controls consisting of high and low standard plasma samples were included on each microtiter plate to detect potential variations between microtitration plates.

Briefly, 96-well microtiter plates (Nunc MaxiSorp, Thermo Fisher Scientific, USA) were coated overnight at 4 °C either with monoclonal antibodies specific for apoB (clone MB47) to capture circulating apoB-containing lipoproteins, or with synthetic MDA-mimotope peptides to detect corresponding autoantibodies. After blocking with phosphate-buffered saline containing 1% bovine serum albumin, diluted plasma samples were added in duplicate and incubated for 2 h at room temperature. Bound antibodies were detected using goat anti-human IgG or IgM secondary antibodies modified with alkaline phosphatase (Jackson ImmunoResearch, USA), followed by chemiluminescent development with Lumi-Phos 530 (Lumigen, Inc, Southfield, MI, USA). Relative light units per 100 ms were measured using a BioTek SYNERGY HTX multi-mode reader luminometer (Winooski, VT, USA).

Results were expressed in arbitrary units relative to the reference plasma as no absolute standards were available. To monitor assay performance, internal controls consisting of high- and low-standard plasma samples were included on each plate. Intra- and inter-assay coefficients of variation were consistently < 10% and < 15%, respectively. Plasma samples were collected in EDTA tubes, stored at −80 °C, and subjected to no more than two freeze–thaw cycles.

### Statistical analysis

Continuous variables were tested for normality by the Kolmogorov–Smirnov test. Data are presented as mean ± standard deviation (SD) and median [interquartile range (IQR)] for parametric and non-parametric data, respectively. For categorical values, frequency counts and percentages were applied. Chi-square (χ^2^) test was performed for interactions between categorical values. Comparisons between baseline and post-treatment measurements were performed using paired statistical tests (paired t-test or Wilcoxon signed-rank test, as appropriate) within each group. The independent sample *t*-test (parametric and non-parametric) was used for the comparison of continuous numeric values between 2 groups. One-way analysis of variance (one-way ANOVA) was performed to assess the difference of the variables of interest between ≥ 2 groups. Multivariate analysis of covariance (MANCOVA) was used for the comparison of continuous numeric values between ≥ 2 groups, adjusting for the baseline value of the variable of interest as well as for key covariates including age, sex, LDL-C, Lp(a), and ASCVD status. Correlation analyses between Lp(a), LDL-C, apoB and IgG apoB-IC at baseline and follow-up as well as between the changes observed over time were performed using Pearson’s correlation coefficient. Analyses were performed for the entire cohort as well as each treatment group. Two-tailed significance was defined as *p* < 0.05. Analyses were performed with the SPSS v21.0 software (SPSS Statistics for Windows, Version 28.0. Armonk, New York, NY, USA: IBM Corp).

## Results

### Baseline characteristics

In total, 70 subjects were included. Specifically, 28 naïve patients received high-intensity statin monotherapy, 31 patients on stable high-intensity statin treatment received add-on ezetimibe and 11 patients on stable high-intensity statin treatment plus ezetimibe received add-on a PCSK9i. The baseline characteristics of the study population are presented in Table 1. Mean age was 51 ± 15 years, 40% were male, 39% were diagnosed with HeFH, 16% had ASCVD, and 36%, 33% and 15% were at very high, high, and moderate cardiovascular risk, respectively. Patients who received add-on ezetimibe ± PCSK9i had significantly increased prevalence of arterial hypertension and type 2 diabetes compared with patients on high-intensity statin monotherapy (*p* = 0.026 and *p* = 0.033, respectively). In addition, patients on triple therapy had significantly increased prevalence of HeFH, ASCVD and chronic kidney disease (CKD) compared with the other 2 groups (*p* = 0.000, *p* = 0.000 and *p* = 0.021, respectively, for the comparison with the high-intensity statin monotherapy group and *p* = 0.000, *p* = 0.000 and *p* = 0.015, respectively, for the comparison with the add-on ezetimibe group).

**Table 1:** Study participant baseline characteristics

	High-intensity statin monotherapy	Add-on ezetimibe to statin	Add-on PCSK9i to statin and ezetimibe	Total sample
Number of patients (N)	28	31	11	70
Gender				
Male	32%	42%	55%	40%
Age (years)	45 (± 16)	54 (± 13)	55 (± 14)	51 (± 15)
Smoking				
Never smoker	64%	58%	60%	61%
Former smoker	11%	16%	30%	16%
Current smoker	25%	26%	10%	23%
Type of high-intensity statin				
Rosuvastatin (median dose 20 mg)	100%	77%	55%	83%
Atorvastatin (median dose 40 mg)	0	23%	45%	17%
Cardiovascular Risk Categories				
Low risk	26%	11%	9%	17%
Intermediate risk	22%	15%	0	15%
High risk	39%	37%	9%	33%
Very high risk	13%	37%	82%	36%
Arterial hypertension	11%	36%^*^	36%	26%
Type 2 diabetes	4%	23%^*^	20%	15%
Heterozygous familial hypercholesterolemia	21%	32%	100%^*#^	39%
Coronary heart disease	0	7%	64%^*#^	13%
Stroke	0	3%	9%	3%
Carotid stenosis	4%	0	9%	3%
Peripheral artery disease	0	0	18%^*#^	3%
Atherosclerotic cardiovascular disease	4%	10%	100%^*#^	16%
Calcific aortic valve stenosis	0	0	9%	1%
Chronic kidney disease	0	0	18%^*#^	3%

Data are presented as N (%). Parametric variables are presented as mean ± SD and non-parametric as median (IQR)

* *p* < 0.05 for the comparison with high-intensity statin monotherapy

# *p* < 0.05 for comparison with high-intensity statin plus ezetimibe

LDL-C; Low-density lipoprotein cholesterol, PCSK9i; proprotein convertase subtilisin/kexin type 9 inhibitors

### Changes in lipid parameters

Across all treatment groups, lipid lowering therapy reduced TC and LDL-C, with add-on PCSK9i demonstrating the largest reductions. ApoB levels declined significantly in all treatment arms by 31%, 24% and 45%, respectively (all *p* = 0.000). No significant change of Lp(a) levels was observed with high-intensity statin and add-on ezetimibe (*p* = 0.24 and* p* = 0.06, respectively). Add-on PCSK9i significantly decreased Lp(a) levels by 43.1% (*p* < 0.05). Detailed data on lipid levels at baseline as well as the effect of lipid lowering medications are presented in Table 2.

**Table 2:** Effect of lipid-lowering treatment on lipid parameters

	Baseline Visit	After 3 Months	% Change within each group
TC, mg/dL, mean (SD)			
High-intensity statin (*n* = 28)	256 (± 38)	177 (± 35)	−31% ^ǂ^
Add-on ezetimibe to statin (*n* = 31)	176 (± 23) ^*^	151 (± 23) ^*^	−14% ^ǂ^
Add-on PCSK9i to statin and ezetimibe (*n* = 11)	217 (± 52) ^*#^	138 (± 43) ^*#^	−36% ^ǂ^
TGs, mg/dL, median (IQR)			
High-intensity statin (*n* = 28)	97 (57;263)	78 (45;371)	−20% ^ǂ^
Add-on ezetimibe to statin (*n* = 31)	105 (44;198)	81 (45;254)	−23%
Add-on PCSK9i to statin and ezetimibe (*n* = 11)	96 (72;270)	79 (44;363)	−18%
HDL-C, mg/dL, mean (SD)			
High-intensity statin (*n* = 28)	60 (± 15)	58 (± 13)	−3% ^ǂ^
Add-on ezetimibe to statin (*n* = 31)	54 (± 16)	54 (± 14)	0
Add-on PCSK9i to statin and ezetimibe (*n* = 11)	52 (± 12)	51 (± 15)	−2%
LDL-C, mg/dL, mean (SD)			
High-intensity statin (*n* = 28)	174 (± 34)	99 (± 27)	−43% ^ǂ^
Add-on ezetimibe to statin (*n* = 31)	101 (± 19) ^*^	79 (± 15) ^*^	−22% ^ǂ^
Add-on PCSK9i to statin and ezetimibe (*n* = 11)	140 (± 45) ^*#^	63 (± 30) ^*#^	−55% ^ǂ^
Lp(a), nmol/L, median (IQR)			
High-intensity statin (*n* = 28)	131.4 (100.4;171.9)	138.7 (111.0;195.5)	+ 5.5%
Add-on ezetimibe to statin (*n* = 31)	128.5 (75.0;184.8)	148.2 (106.6;179.9)	+ 15.3%
Add-on PCSK9i to statin and ezetimibe (*n* = 11)	139.6 (75.0;278.9)	84.8 (39.5;210.9)	−43.1% ^ǂ^
ApoB, mg/dL, mean (SD)			
High-intensity statin (*n* = 28)	102 (± 27)	70 (± 18)	−31% ^ǂ^
Add-on ezetimibe to statin (*n* = 31)	85 (± 26)^*^	65 (± 13)	−24% ^ǂ^
Add-on PCSK9i to statin and ezetimibe (*n* = 11)	107 (± 22) ^#^	59 (± 19)	−45% ^ǂ^

ǂ *p* < 0.05 for the comparison within each group

* *p* < 0.05 for the comparison with patients treated with high-intensity statin

# *p* < 0.05 for the comparison with patients treated with high-intensity statin plus ezetimibe

TC; Total Cholesterol, TGs; Triglycerides, HDL-C; High-density Lipoprotein Cholesterol, LDL-C; Low-density Lipoprotein Cholesterol, Lp(a); lipoprotein(a), ApoB; apolipoprotein B-100, PCSK9i; proprotein convertase subtilisin/kexin type 9 inhibitors

### Changes in IgG and IgM apoB-IC and MDA-mimotope

Significant decreases in IgG apoB-IC levels of 18.3%, 17.5%, and 25.5% were noted in all groups, respectively (all *p* < 0.05) (Table 3). In all treatment groups, IgM apoB-IC, IgG and IgM anti-MDA-mimotope levels did not significantly change. Baseline values and follow-up measurements for these biomarkers are detailed in Table 3.

**Table 3:** Effect of lipid-lowering treatment on malondialdehyde-modified apoB and apoB-immune complexes

	Baseline Visit	After 3 Months	% Change within each group
IgG apoB-IC, RLU, median (IQR)			
High-intensity statin (*n* = 28)	4326.5 (3821.5;5806.0)	3536.0 (2650.0;5112.0)	−18.3% ^ǂ^
Add-on ezetimibe to statin (*n* = 31)	4820.0 (3378.0;6455.0)	3976.0 (3099.0;4904.0)	−17.5% ^ǂ^
Add-on PCSK9i to statin and ezetimibe (*n* = 11)	4895.0 (3598.0;5926.0)	3646.0 (2589.0;7838.0)	−25.5% ^ǂ^
IgM apoB-IC, RLU, median (IQR)			
High-intensity statin (*n* = 28)	2865.5 (2199.5; 3957.3)	3345.5 (2520.8; 4574.8)	+ 16.8%
Add-on ezetimibe to statin (*n* = 31)	3297.0 (1806.0; 4364.0)	3236.0 (1866.0; 4806.0)	−1.9%
Add-on PCSK9i to statin and ezetimibe (*n* = 11)	3312.0 (2422.0; 4060.0)	3818.0 (2686.0; 5319.0)	+ 15.3%
IgG anti-MDA-mimotope, RLU, median (IQR)			
High-intensity statin (*n* = 28)	3603.5 (1803.5; 4942.8)	3023.5 (1725.0; 5115.8)	−16.1%
Add-on ezetimibe to statin (*n* = 31)	2996.0 (2043.0; 4201.0)	2831.0 (1793.0; 3992.0)	−5.5%
Add-on PCSK9i to statin and ezetimibe (*n* = 11)	2692.0 (1593.0; 3915.0)	2573.0 (1573.0; 3227.0)	−4.4%
IgM anti-MDA-mimotope, RLU, median (IQR)			
High-intensity statin (*n* = 28)	4313.0 (3281.3; 7049.0)	5774.5 (4035.8; 7617.3)	+ 33.9%
Add-on ezetimibe to statin (*n* = 31)	5349.0 (2729.0; 8177.0)	4606.0 (2425.0; 6806.0)	−13.9%
Add-on PCSK9i to statin and ezetimibe (*n* = 11)	4740.0 (3119.0; 6566.0)	4753.0 (2725.0; 7672.0)	+ 0.3%

ǂ p < 0.05 for the comparison within each group

*RLU* Relative Light Units, *MDA-apoB* malondialdehyde-modified human apolipoprotein B-100, *IgG apoB-IC* IgG apolipoproteinB-100-containing immune complexes, *IgM apoB-IC* IgM apolipoproteinB-100-containing immune complexes, *IgG MDA-mimotope* IgG autoantibodies to a malondialdehyde mimotope, *IgM MDA-mimotope* IgM autoantibodies to a malondialdehyde mimotope, *PCSK9i* proprotein convertase subtilisin/kexin type 9 inhibitors

### Correlation analyses

**Fig. 1:**
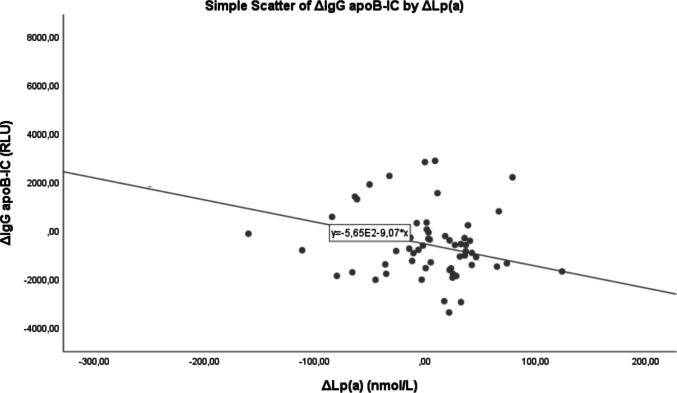
Correlation between changes in IgG apoB-IC and Lp(a) from baseline to follow-up visit (after 3 months). Abbreviations: IgG apoB-IC; IgG apolipoproteinB-100-containing immune complexes, Lp(a); lipoprotein(a), ΔLp(a); Change in Lp(a), ΔIgG apoB-IC; Change in IgG apoB-IC. Legend: Scatterplot shows individual data points with a fitted regression line and 95% confidence interval. Spearman’s *r* = −0.3, *p* = 0.017

In the total population, correlation analyses at baseline and follow-up revealed no significant correlations between IgG apoB-IC with apoB, Lp(a) or LDL-C. Correlation analyses based on changes from baseline to follow-up revealed that changes of IgG apoB-IC were significantly associated with changes in Lp(a) (*r* = −0.3, *p* = 0.017) (Fig. 1). When analyzed by treatment group, similar correlation patterns were observed (data not shown).

## Discussion

This study investigated, for the first time, the impact of lipid-lowering therapies on circulating levels of IgG and IgM autoantibodies in patients with Lp(a) levels ≥ 75 nmol/L. High-intensity statins, add-on ezetimibe and add-on PCSK9i, were associated with significant reductions in IgG apoB-IC levels, while IgM apoB-IC and both IgG and IgM anti-MDA-mimotope levels remained unchanged.

In patients treated with high-intensity statins, we observed a significant reduction in IgG apoB-IC autoantibodies, while IgM apoB-IC autoantibody levels remained unchanged. These findings suggest that statins may exert selective immunomodulatory effects, particularly on the IgG response to apoB-immune complexes. The reduction in IgG apoB-IC levels with high-intensity statin therapy may reflect statin-associated attenuation of chronic immune activation or reduced formation of circulating immune complexes. This is supported by previous studies indicating that intensive statin therapy can reduce levels of circulating immune complexes and immunogenic lipoprotein modifications[7]. The absence of significant changes in IgM autoantibody levels aligns with prior evidence suggesting that these natural antibodies are predominantly involved in maintaining immune homeostasis and are less susceptible to modulation by short-term pharmacologic interventions [28].

Among individuals receiving ezetimibe added to high-intensity statin therapy, we also observed a significant reduction in IgG apoB IC autoantibodies and no notable changes in IgM apoB-IC antibody levels. Although data specifically addressing ezetimibe effect on these autoantibodies are limited, it can be assumed that the significant drop in IgG apoB‑IC may reflect additive benefits of ezetimibe in lowering of oxidative neoepitopes.

When PCSK9i were added to statin and ezetimibe therapy we observed a significant reduction in IgG apoB-IC autoantibodies, but no significant change in IgM apoB-IC autoantibodies levels. Mechanistic studies indicate that PCSK9 inhibition dampens monocyte activation and inflammatory phenotype but does not necessarily reduce antibody responses or oxidation-specific biomarkers [29, 30].

**Fig. 2:**
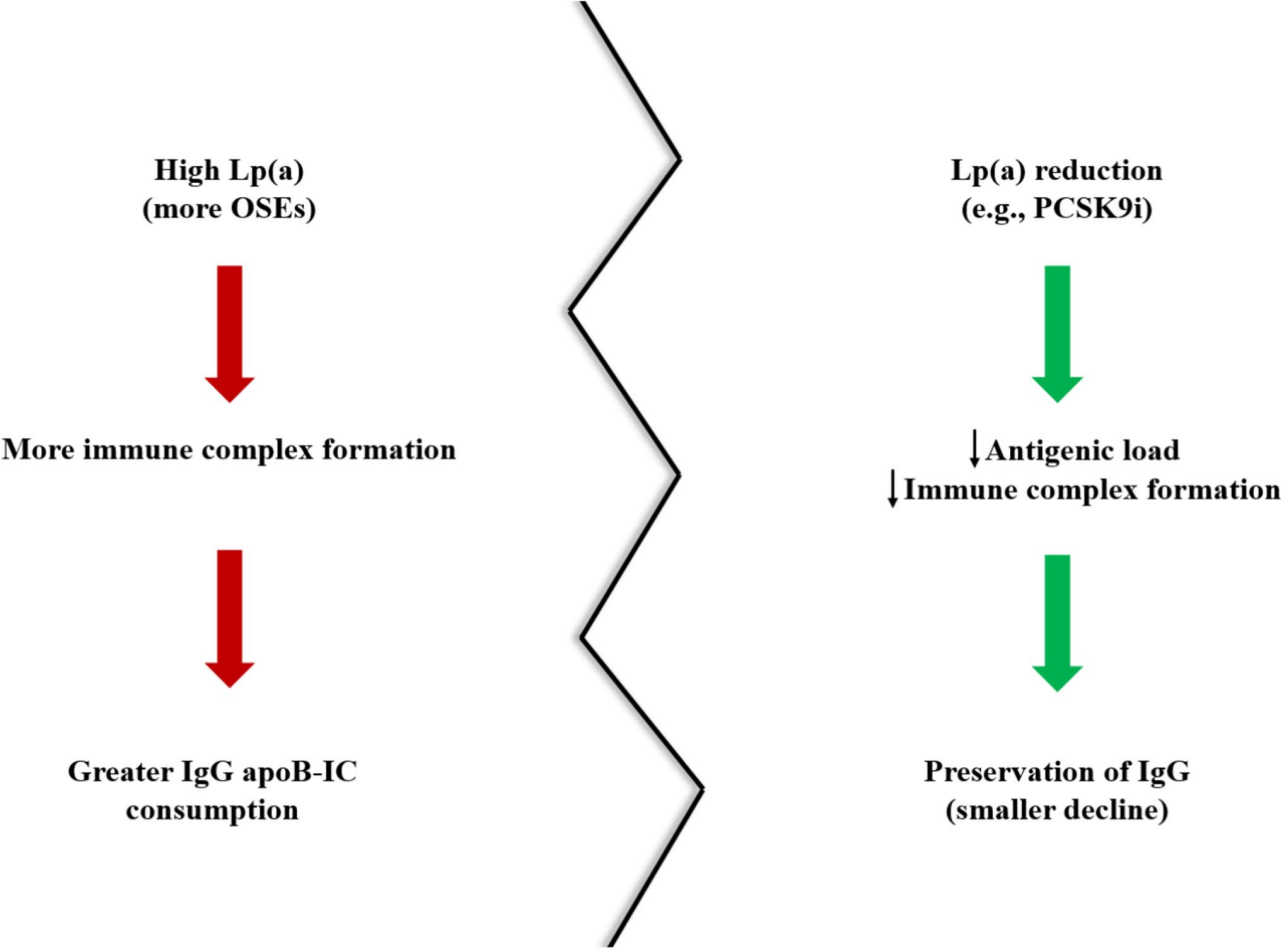
Potential explanation illustrating the relationship between lipoprotein(a) reduction and IgG apoB-IC decline. Abbreviations: IgG apoB-IC; IgG apolipoproteinB-100-containing immune complexes, Lp(a); lipoprotein(a), OSEs; oxidation-specific epitopes, PCSK9i; proprotein convertase subtilisin/kexin type 9 inhibitor

Correlation analyses showed that individuals who experienced larger changes in Lp(a) tended to show smaller changes in IgG apoB-IC levels. This suggests that when Lp(a) -a major carrier of oxidation-specific epitopes- declines substantially, the antigenic stimulus for antibody production diminishes, stabilizing IgG responses. Conversely, in patients with smaller Lp(a) reductions, continued exposure to oxidation-specific epitopes may sustain immune complex formation, leading to more pronounced decreases in circulating IgG apoB-IC due to ongoing antibody-antigen complexing and clearance (Fig. 2). Conversely, in individuals whose Lp(a) levels increased during follow-up, the enhanced availability of Lp(a)-associated oxidation-specific epitopes could potentially sustain or even augment immune stimulation. However, in our study the magnitude of Lp(a) increases was small, and therefore we did not observe a corresponding rise in IgG apoB-IC levels. Taken together, these observations suggest that both the direction and magnitude of Lp(a) change may influence circulating immune complex dynamics. These findings support the concept that apoB-containing lipoproteins [such as Lp(a)] serve as antigenic carriers of oxidation-specific epitopes that modulate adaptive immune responses [31].

With respect to MDA-mimotope antibodies, neither high-intensity statins, nor add-on ezetimibe, nor add-on PCSK9i altered IgG or IgM responses to MDA-mimotope peptides. Data on these antibodies are limited, but they are thought to reflect recognition of specific MDA-related epitopes within oxLDL. Given that reductions in oxLDL with ezetimibe plus statins have been documented [32, 33], one might expect parallel effects on MDA-mimotope responses. However, the unchanged antibody titers in our study may indicate that either longer treatment duration is needed, or that these epitopes are less immunogenic and thus resistant to modulation by lipid lowering.

Several limitations should be acknowledged. First, the follow-up period was relatively short, potentially limiting the detection of long-term effects. Second, the absence of randomization and placebo control introduces the possibility of confounding and bias. Third, the sample size, particularly in the PCSK9i group, was small, reducing statistical power and limiting generalizability. Consequently, these findings should be considered hypothesis-generating and interpreted cautiously. Additionally, important baseline differences existed among treatment groups, including a higher prevalence of ASCVD, HeFH, and CKD in patients receiving add-on PCSK9i. Despite adjustment, these differences may have influenced the biomarker response to treatment. Furthermore, patients receiving add-on ezetimibe or add-on PCSK9i were already on a stable lipid-lowering regimen prior to treatment intensification. Thus, our findings reflect the additive rather than the isolated effects of these agents.

## Conclusions

In individuals with elevated Lp(a) levels, high-intensity statins, add-on ezetimibe and add-on PCSK9i reduced IgG apoB-IC. No significant changes were observed in IgM apoB-IC or anti-MDA-mimotope levels. The clinical implications of these findings remain uncertain and warrants further investigation.

## Data Availability

The datasets generated and/or analyzed during the current study are not publicly available due to patient privacy and institutional restrictions but are available from the corresponding author on reasonable request.

## References

[1] Ajoolabady A, Pratico D, Lin L, Mantzoros CS, Bahijri S, Tuomilehto J, et al. Inflammation in atherosclerosis: pathophysiology and mechanisms. Cell Death Dis [Internet]. 2024 Nov 1 [cited 2025 Oct 2];15(11):817. Available from: https://pmc.ncbi.nlm.nih.gov/articles/PMC11555284/10.1038/s41419-024-07166-8 PMC11555284 39528464

[2] Fredrikson GN, Hedblad B, Berglund G, Alm R, Ares M, Cercek B, et al. Identification of immune responses against aldehyde-modified peptide sequences in apoB associated with cardiovascular disease. Arterioscler Thromb Vasc Biol [Internet]. 2003 May 1 [cited 2025 Aug 5];23(5):872–8. Available from: https://pubmed.ncbi.nlm.nih.gov/12649091/10.1161/01.ATV.0000067935.02679.B0 12649091

[3] Nègre-Salvayre A, Salvayre R. Reactive Carbonyl Species and Protein Lipoxidation in Atherogenesis. Antioxidants 2024, Vol 13, Page 232 [Internet]. 2024 Feb 14 [cited 2025 Oct 2];13(2):232. Available from: https://www.mdpi.com/2076-3921/13/2/232/htm 10.3390/antiox13020232 PMC10886358 38397830

[4] Orekhov AN, Bobryshev Y V., Sobenin IA, Melnichenko AA, Chistiakov DA. Modified Low Density Lipoprotein and Lipoprotein-Containing Circulating Immune Complexes as Diagnostic and Prognostic Biomarkers of Atherosclerosis and Type 1 Diabetes Macrovascular Disease. International Journal of Molecular Sciences 2014, Vol 15, Pages 12807–12841 [Internet]. 2014 Jul 21 [cited 2025 Jul 28];15(7):12807–41. Available from: https://www.mdpi.com/1422-0067/15/7/12807/htm 10.3390/ijms150712807 PMC4139876 25050779

[5] Grönwall C, Amara K, Hardt U, Krishnamurthy A, Steen J, Engström M, et al. Autoreactivity to malondialdehyde-modifications in rheumatoid arthritis is linked to disease activity and synovial pathogenesis. J Autoimmun [Internet]. 2017 Nov 1 [cited 2025 Jul 28];84:29–45. Available from: http://arxiv.org/abs/1710.10861 10.1016/j.jaut.2017.06.004 28647488

[6] Luquain-Costaz C, Delton I. Oxysterols in Vascular Cells and Role in Atherosclerosis. Adv Exp Med Biol [Internet]. 2024 Sep 24 [cited 2025 Jul 28];1440:213–29. Available from: http://arxiv.org/abs/2409.15874 10.1007/978-3-031-43883-7_11 38036882

[7] Hartley A, Pradeep M, Van den Berg V, Khan AHA, Shah HA, Allaf M, et al. Depletion of Homeostatic Antibodies against MalondialdehydeModified Low-Density Lipoprotein Correlates with Adverse Events in Major Vascular Surgery. Antioxidants [Internet]. 2022 Feb 1 [cited 2025 Jul 28];11(2):271. Available from: https://www.mdpi.com/2076-3921/11/2/271/htm 10.3390/antiox11020271 PMC8868419 35204154

[8] Marchini T, Malchow S, Caceres L, El Rabih AAH, Hansen S, Mwinyella T, et al. Circulating Autoantibodies Recognizing Immunodominant Epitopes From Human Apolipoprotein B Associate With Cardiometabolic Risk Factors, but Not With Atherosclerotic Disease. Front Cardiovasc Med [Internet]. 2022 Apr 11 [cited 2025 Jul 28];9:826729. Available from: www.frontiersin.org 10.3389/fcvm.2022.826729 PMC9035541 35479271

[9] Asciutto G, Wigren M, Fredrikson GN, Mattisson IY, Grönberg C, Alm R, et al. Apolipoprotein B-100 antibody interaction with atherosclerotic plaque inflammation and repair processes. Stroke [Internet]. 2016 [cited 2025 Jul 28];47(4):1140–3. Available from: 10.1161/STROKEAHA.116.012677 10.1161/STROKEAHA.116.012677 26965851

[10] Samal SK, Leander K, Vikström M, Griesbaum L, de Faire U, Frostegård J. Antibodies against malondialdehyde among 60-year-olds: prediction of cardiovascular disease. Sci Rep [Internet]. 2023 Dec 1 [cited 2025 Jul 28];13(1):1–9. Available from: https://www.nature.com/articles/s41598-023-42264-1 10.1038/s41598-023-42264-1 PMC10495339 37697019

[11] Prasad A, Clopton P, Ayers C, Khera A, De Lemos JA, Witztum JL, et al. Relationship of Autoantibodies to MDA-LDL and ApoB-Immune Complexes to Sex, Ethnicity, Subclinical Atherosclerosis and Cardiovascular Events. Arterioscler Thromb Vasc Biol [Internet]. 2017 Jun 1 [cited 2025 Jul 28];37(6):1213. Available from: https://pmc.ncbi.nlm.nih.gov/articles/PMC5500201/10.1161/ATVBAHA.117.309101 PMC5500201 28473443

[12] Amir S, Hartvigsen K, Gonen A, Leibundgut G, Que X, Jensen-Jarolim E, et al. Peptide mimotopes of malondialdehyde epitopes for clinical applications in cardiovascular disease. J Lipid Res [Internet]. 2012 Jul [cited 2025 Oct 14];53(7):1316. Available from: https://pmc.ncbi.nlm.nih.gov/articles/PMC3371243/10.1194/jlr.M025445 PMC3371243 22508944

[13] McLeod O, Silveira A, Fredrikson GN, Gertow K, Baldassarre D, Veglia F, et al. Plasma autoantibodies against apolipoprotein B-100 peptide 210 in subclinical atherosclerosis. Atherosclerosis [Internet]. 2014 Jan 1 [cited 2025 Jul 28];232(1):242–8. Available from: https://www.sciencedirect.com/science/article/abs/pii/S0021915013006928?utm_source=chatgpt.com 10.1016/j.atherosclerosis.2013.11.041 24401246

[14] Fagerberg B, Gullberg UP, Alm R, Nilsson J, Fredrikson GN. Circulating Autoantibodies against the Apolipoprotein B-100 Peptides p45 and p210 in Relation to the Occurrence of Carotid Plaques in 64-Year-Old Women. PLoS One [Internet]. 2015 Mar 13 [cited 2025 Jul 28];10(3):e0120744. Available from: https://journals.plos.org/plosone/article?id=10.1371/journal.pone.0120744 10.1371/journal.pone.0120744 PMC4358991 25768285

[15] Yamamoto H, Kawamura M, Kochi I, Imai M, Murata Y, Suzuki T, et al. Serum Anti-Apo B Antibody Level as Residual CVD Marker in DM Patients under Statin Treatment. J Atheroscler Thromb [Internet]. 2019 [cited 2025 Jul 28];26(10):931. Available from: https://pmc.ncbi.nlm.nih.gov/articles/PMC6800396/10.5551/jat.46797 PMC6800396 30867375

[16] Tsimikas S, Witztum JL. The role of oxidized phospholipids in mediating lipoprotein(a) atherogenicity. Curr Opin Lipidol [Internet]. 2008 Aug [cited 2025 Aug 5];19(4):369–77. Available from: https://journals.lww.com/co-lipidology/fulltext/2008/08000/the_role_of_oxidized_phospholipids_in_mediating.8.aspx 10.1097/MOL.0b013e328308b622 18607184

[17] Bergmark C, Dewan A, Orsoni A, Merki E, Miller ER, Shin MJ, et al. A novel function of lipoprotein [a] as a preferential carrier of oxidized phospholipids in human plasma. J Lipid Res [Internet]. 2008 Oct 1 [cited 2025 Aug 5];49(10):2230–9. Available from: https://www.jlr.org/action/showFullText?pii=S0022227520346460 10.1194/jlr.M800174-JLR200 18594118

[18] Leibundgut G, Scipione C, Yin H, Schneider M, Boffa MB, Green S, et al. Determinants of binding of oxidized phospholipids on apolipoprotein (a) and lipoprotein (a). J Lipid Res [Internet]. 2013 Oct [cited 2025 Aug 5];54(10):2815. Available from: https://pmc.ncbi.nlm.nih.gov/articles/PMC3770094/10.1194/jlr.M040733 PMC3770094 23828779

[19] Mach F, Koskinas KC, Roeters van Lennep JE, Tokgözoğlu L, Badimon L, Baigent C, et al. 2025 Focused Update of the 2019 ESC/EAS Guidelines for the management of dyslipidaemias: Developed by the task force for the management of dyslipidaemias of the European Society of Cardiology (ESC) and the European Atherosclerosis Society (EAS). Eur Heart J [Internet]. 2025 Aug 29 [cited 2025 Oct 2]; Available from: 10.1093/eurheartj/ehaf190

[20] Koutsogianni AD, Barkas F, Tellis C, Tselepis A, Liamis G, Tsimikas S, et al. Effect of lipid-lowering medications on oxidized phospholipids in individuals with elevated lipoproteiN(a). Atherosclerosis Plus [Internet]. 2025 Sep 4 [cited 2025 Sep 7]; Available from: https://linkinghub.elsevier.com/retrieve/pii/S2667089525000239 10.1016/j.athplu.2025.09.003 PMC12455066 40994740

[21] Katsiki N, Filippatos T, Vlachopoulos C, Panagiotakos D, Milionis H, Tselepis A, et al. Executive summary of the Hellenic Atherosclerosis Society guidelines for the diagnosis and treatment of dyslipidemias - 2023. Atherosclerosis Plus [Internet]. 2024 Mar 1 [cited 2025 Jul 28];55:74. Available from: https://pmc.ncbi.nlm.nih.gov/articles/PMC10901915/10.1016/j.athplu.2024.01.004 PMC10901915 38425675

[22] Marcovina SM, Navabi N, Allen S, Gonen A, Witztum JL, Tsimikas S. Development and validation of an isoform-independent monoclonal antibody–based ELISA for measurement of lipoprotein(a). J Lipid Res [Internet]. 2022 Aug 1 [cited 2025 Jul 28];63(8):100239. Available from: https://pmc.ncbi.nlm.nih.gov/articles/PMC9352967/10.1016/j.jlr.2022.100239 PMC9352967 35688187

[23] Bevan RJ, Durand MF, Hickenbotham PT, Kitas GD, Patel PR, Podmore ID, et al. Validation of a novel ELISA for measurement of MDA-LDL in human plasma. Free Radic Biol Med [Internet]. 2003 Sep 1 [cited 2025 Jul 28];35(5):517–27. Available from: https://www.sciencedirect.com/science/article/abs/pii/S0891584903003599?utm_source=chatgpt.com 10.1016/s0891-5849(03)00359-9 12927601

[24] Tsimikas S, Willeit P, Willeit J, Santer P, Mayr M, Xu Q, et al. Oxidation-specific biomarkers, prospective 15-year cardiovascular and stroke outcomes, and net reclassification of cardiovascular events. J Am Coll Cardiol [Internet]. 2012 Nov 20 [cited 2025 Sep 7];60(21):2218–29. Available from: 10.1016/j.jacc.2012.08.979 10.1016/j.jacc.2012.08.979 23122790

[25] Tsimikas S, Bergmark C, Beyer RW, Patel R, Pattison J, Miller E, et al. Temporal increases in plasma markers of oxidized low-density lipoprotein strongly reflect the presence of acute coronary syndromes. J Am Coll Cardiol [Internet]. 2003 Feb 5 [cited 2025 Sep 7];41(3):360–70. Available from: 10.1016/S0735-1097(02)02769-9 10.1016/s0735-1097(02)02769-9 12575961

[26] Tsimikas S, Palinski W, Witztum JL. Circulating autoantibodies to oxidized LDL correlate with arterial accumulation and depletion of oxidized LDL in LDL receptor-deficient mice. Arterioscler Thromb Vasc Biol [Internet]. 2001 [cited 2025 Sep 7];21(1):95–100. Available from: 10.1161/01.ATV.21.1.95 10.1161/01.atv.21.1.95 11145939

[27] Hörkkö S, Bird DA, Miller E, Itabe H, Leitinger N, Subbanagounder G, et al. Monoclonal autoantibodies specific for oxidized phospholipids or oxidized phospholipid–protein adducts inhibit macrophage uptake of oxidized low-density lipoproteins. Journal of Clinical Investigation [Internet]. 1999 [cited 2025 Sep 7];103(1):117. Available from: https://pmc.ncbi.nlm.nih.gov/articles/PMC407862/10.1172/JCI4533 PMC407862 9884341

[28] Dunér P, To F, Berg K, Alm R, Björkbacka H, Engelbertsen D, et al. Immune responses against aldehyde-modified laminin accelerate atherosclerosis in Apoe−/− mice. Atherosclerosis [Internet]. 2010 Oct 1 [cited 2025 Jul 31];212(2):457–65. Available from: https://www.sciencedirect.com/science/article/abs/pii/S0021915010005563?utm_source=chatgpt.com 10.1016/j.atherosclerosis.2010.07.014 20810111

[29] Bernelot Moens SJ, Neele AE, Kroon J, Van Der Valk FM, Van Den Bossche J, Hoeksema MA, et al. PCSK9 monoclonal antibodies reverse the pro-inflammatory profile of monocytes in familial hypercholesterolaemia. Eur Heart J [Internet]. 2017 May 21 [cited 2025 Jul 31];38(20):1584–93. Available from: https://pubmed.ncbi.nlm.nih.gov/28329114/10.1093/eurheartj/ehx002 28329114

[30] Wu NQ, Shi HW, Li JJ. Proprotein Convertase Subtilisin/Kexin Type 9 and Inflammation: An Updated Review. Front Cardiovasc Med [Internet]. 2022 Feb 18 [cited 2025 Jul 31];9:763516. Available from: www.frontiersin.org 10.3389/fcvm.2022.763516 PMC8894439 35252378

[31] Gerdes N, Klingenberg R (2024Aug) Mini-Review: Immunogenic epitopes in apolipoprotein B-100 for atheroprotective immunization. Front Cardiovasc Med 15(11):1448664 10.3389/fcvm.2024.1448664 PMC11357920 39211769

[32] Jamialahmadi T, Baratzadeh F, Reiner Ž, Simental-Mendía LE, Xu S, Susekov A V., et al. The Effects of Statin Dose, Lipophilicity, and Combination of Statins plus Ezetimibe on Circulating Oxidized Low-Density Lipoprotein Levels: A Systematic Review and Meta-Analysis of Randomized Controlled Trials. Mediators Inflamm [Internet]. 2021 [cited 2025 Jul 31];2021:9661752. Available from: https://pmc.ncbi.nlm.nih.gov/articles/PMC8437664/10.1155/2021/9661752 PMC8437664 34526854

[33] Azar RR, Badaoui G, Sarkis A, Azar M, Aydanian H, Harb S, et al. Effect of ezetimibe/atorvastatin combination on oxidized low density lipoprotein cholesterol in patients with coronary artery disease or coronary artery disease equivalent. American Journal of Cardiology [Internet]. 2010 Jul 15 [cited 2025 Jul 31];106(2):193–7. Available from: https://pubmed.ncbi.nlm.nih.gov/20599002/10.1016/j.amjcard.2010.03.016 20599002

